# Primary urethral small cell melanoma with neuroendocrine differentiation: a case report

**DOI:** 10.1186/s43046-020-00051-3

**Published:** 2020-10-12

**Authors:** Tripti Nakra, Rohit Dadhwal, Rishi Nayyar, Sameer Rastogi, Aanchal Kakkar, Mehar Chand Sharma, Rajni Yadav

**Affiliations:** 1 Department of Pathology, All India Institute of Medical Sciences, Ansari Nagar, New Delhi, 110029 India; 2 Department of Urology, All India Institute of Medical Sciences, Ansari Nagar, New Delhi, 110029 India; 3 Department of Medical Oncology, All India Institute of Medical Sciences, Ansari Nagar, New Delhi, 110029 India

**Keywords:** Immunohistochemistry, Melanoma, Neuroendocrine, Neurosecretory granules, Urethral

## Abstract

**Background:**

Primary malignant melanoma of the female urethra is an exceedingly rare tumor. It represents 0.2% of all malignant melanomas. Divergent differentiation towards non-melanocytic lineages has not been reported in urethral melanoma.

**Case presentation:**

We report a rare case of neuroendocrine differentiation in a large primary small cell malignant melanoma involving the urethra, in a 62-year-old lady, who presented with obstructive urinary symptoms. Clinical and radiological workup revealed a large urethral mass with liver and lymph nodal metastases. A biopsy was performed from the urethral and liver lesions which showed poorly differentiated tumor cells with small cell morphology and presence of melanin pigment. These cells were immunopositive for melanocytic and neuroendocrine markers. Ultrastructural examination showed presence of melanosomes and neurosecretory granules in the tumor cells.

**Conclusions:**

Although malignant melanoma with neuroendocrine differentiation is exceptionally rare, it needs to be recognized among the other well-known variants of malignant melanoma.

## Background

Primary malignant melanoma (MM) of the lower genitourinary tract is an uncommon malignancy with the distal urethra being the most common site of origin. It is extremely rare in females and occurs mostly in the sixth to seventh decade of life [1–3]. This tumor frequently presents as a polypoidal mass and clinically mimics a urethral polyp or caruncle. Due to its behavior of rapid progression and spread, the diagnosis may be delayed as the local tumor remains small in size and may not produce much symptoms. At the time of diagnosis, the urethral tumor carries a worse prognosis than its cutaneous counterpart [1]. The presence of divergent differentiation toward epithelial, smooth muscle, rhabdomyoblastic, or neuroendocrine lineage is an unusual phenomenon in MM [4]. MM with neuroendocrine differentiation involving the female urethra as the primary site has not been documented in the literature till date. Here, we report a case of primary small cell melanoma of female urethra with neuroendocrine differentiation. Our case exhibited an almost complete complement of melanocytic and neuroendocrine markers by both immunohistochemistry and ultrastructural examination.

## Case presentation

**Fig. 1:**
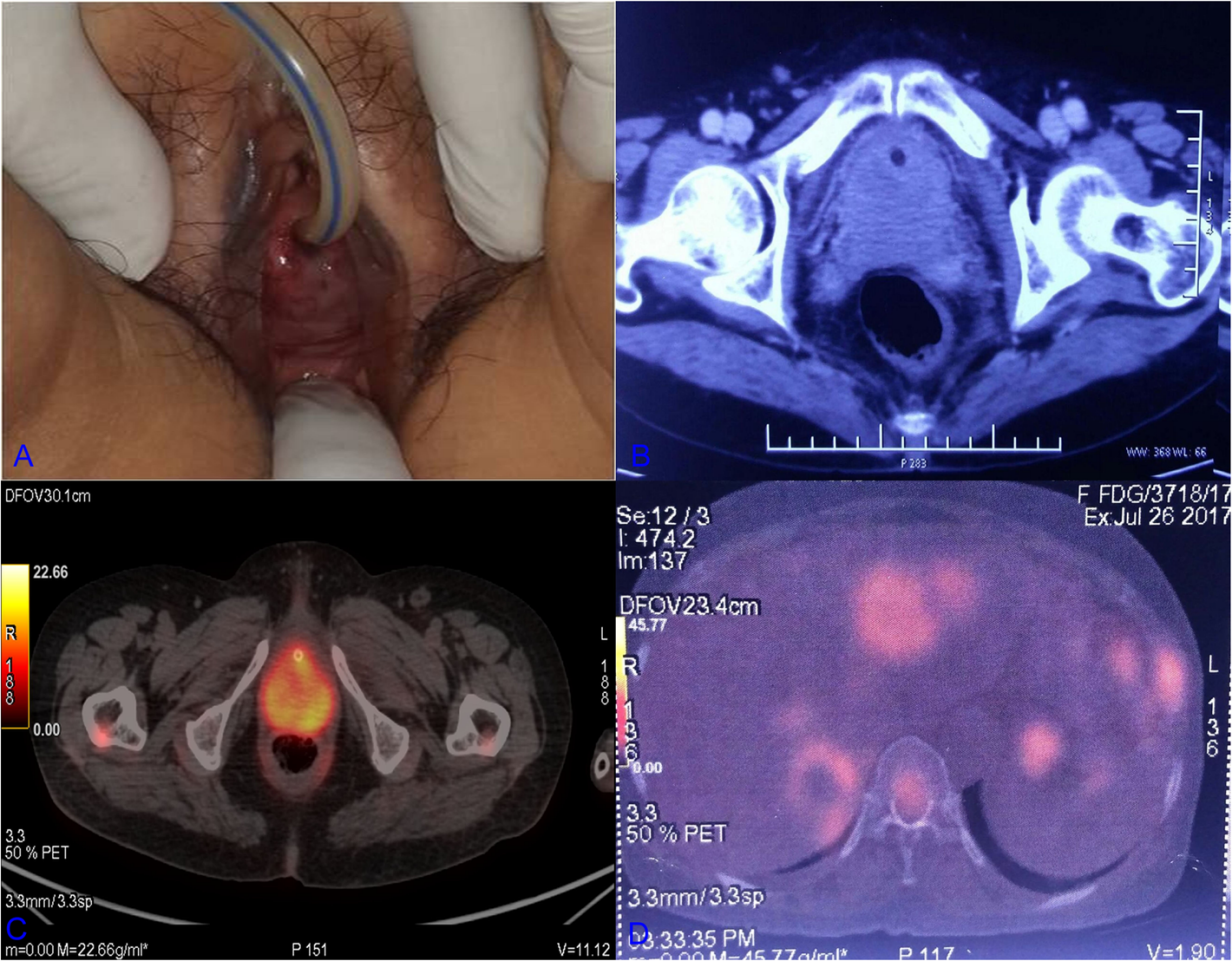
Clinicoimaging findings: (**a**) Local examination revealed a mass with bluish tinge stretching the external urethral meatus. A catheter was placed which drained clear urine. (**b**) Computed tomography scan showed a large urethral mass which was further highlighted with (**c**) positron emission tomography scan. (**d**) In addition, multiple metastatic lesions were seen in the liver

A 62-year-old post-menopausal lady, without any previous co-morbidity, presented to our hospital with acute urinary retention, antecedent history of obstructive lower urinary tract symptoms, and occasional mild initial hematuria for the last 3 months. History of undocumented weight loss and loss of appetite was also present. The abdominal examination was normal. Per vaginal and speculum examination revealed a large mildly tender mass with a soft bluish tinge in the distal third of the vagina that was stretching the external urethral meatus. The mass extended across the entire length of the urethra and the overlying mucosa was normal. There was no bleed to touch or pus discharge. A catheter was placed to relieve the urinary retention and drained clear urine (Fig. 1a).

Multiphasic computed tomography (CT) scan of the abdomen and pelvis (Fig. 1b) showed 43 × 50 mm mass in the urethra bulging into the lumen of the urinary bladder along with bilateral hydroureteronephrosis. Hypodense lesions were also seen in segments IIa, III, V, and VI of the liver with the largest lesion (41 × 40 mm) present in the left lobe. Positron emission tomography (PET) CT scan (Fig. 1c and d) confirmed urethral mass, multiple large liver metastatic lesions, and additionally showed multiple enlarged cervical, supraclavicular, paratracheal, subcarinal, anterior diaphragmatic, internal mammary, retrosternal, and bilateral inguinal lymph nodes with increased tracer uptake. In addition, FDG avid paraaortic, aortocaval, paracaval, retrocaval, and mesenteric lymph nodes were also noted. The pancreas appeared bulky with nodular lesions and foci of increased uptake. Cystourethroscopy revealed a mass effect involving the whole length of the urethra. Urethral mucosa, urinary bladder, and bilateral ureteric orifices were normal. A trucut biopsy was taken from the urethral mass.

**Fig. 2:**
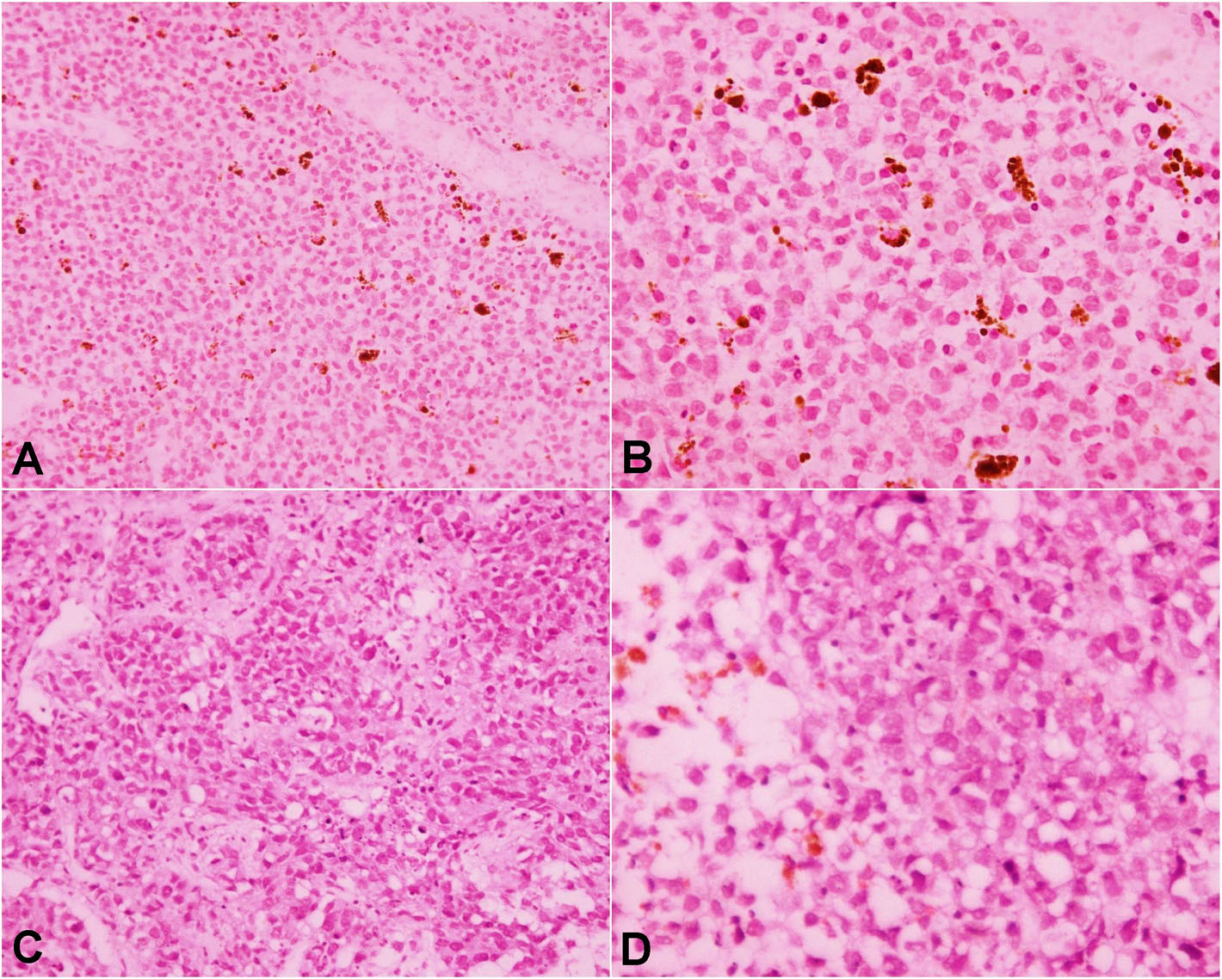
Histopathological examination of urethral small cell melanoma with neuroendocrine differentiation: Biopsy from the urethral mass showed tumor cells arranged in a solid pattern (**a**, H&E, ×200). The tumor cells had high nucleocytoplasmic ratio, moderately pleomorphic nuclei with conspicuous nucleoli at places, and melanin granules in the cytoplasm (**b**, H&E, ×400). Biopsy from the liver lesion also showed a similar tumor. Frequent apoptoses and mitoses were seen (H&E; ×200, **c**; and ×400, **d**)

**Fig. 3:**
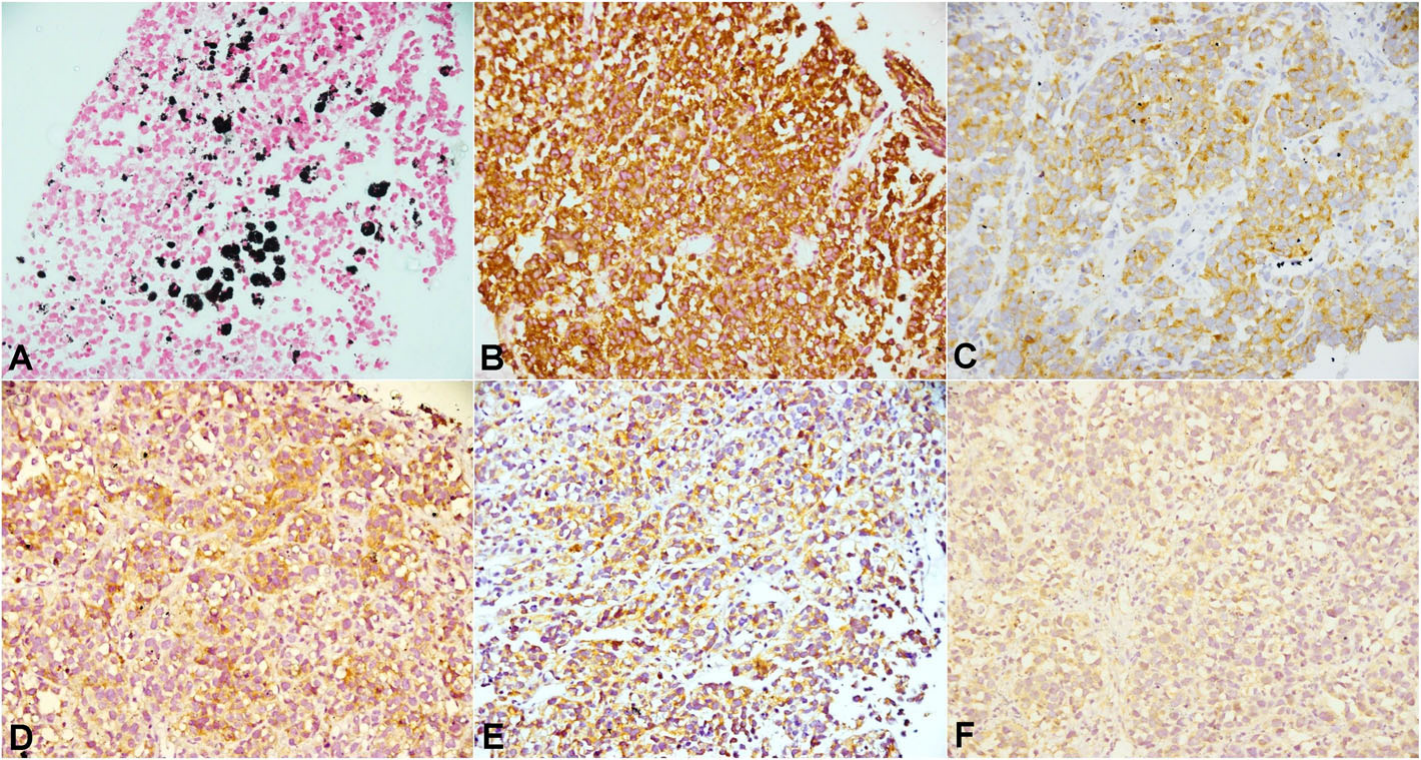
Histochemical and immunohistochemical findings: Fontana-Masson stain highlighted the black stained melanin granules (**a**, FM, ×200). The tumor cells were immunopositive for HMB-45 (**b**), Melan-A (**c**), CD56 (**d**), chromogranin (**e**), and synaptophysin (**f**); ×200

**Fig. 4:**
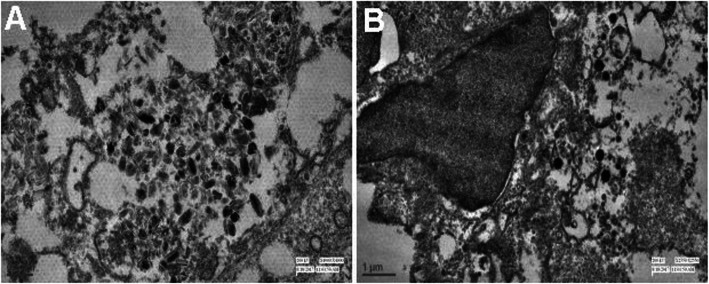
Electron microscopic features: Ultrastructural examination revealed the presence of melanosomes (**a**) and neurosecretory granules (**b**) in the cytoplasm of tumor cells

Histopathological examination showed a tumor with solid sheet pattern with few intervening small blood vessels (Fig. 2a-c). The tumor was composed of small cells with scant pale cytoplasm and ill-defined cell borders, high nucleo-cytoplasmic ratio, and moderate nuclear pleomorphism. Focally, nuclear molding and smudging were also identified. Frequent apoptoses and mitoses including atypical forms were appreciated (Fig. 2d). Some of these cells showed conspicuous eosinophilic nucleoli at places and the presence of melanin pigment in the cytoplasm which was highlighted on Fontana-Masson stain (Fig. 3a). As it was a trucut biopsy of mass, overlying mucosa was not included in the section to comment upon the junctional activity. A panel of immunohistochemical stains (Fig. 3b-f) were performed which demonstrated tumor cell immunopositivity for HMB-45 (1:250 dilution, mouse monoclonal antibody, clone HMB-45, Bio-SB, USA), Melan-A (1:250 dilution, mouse monoclonal antibody, clone A103, Bio-SB, USA), S-100 (1:400 dilution, rabbit polyclonal antibody, Dako, USA), synaptophysin (1:250 dilution, rabbit monoclonal antibody, clone EP158, Bio-SB, USA), chromogranin (1:200 dilution, mouse monoclonal antibody, clone LK2H10, SycTek, USA), and CD56 (1:200 dilution, mouse monoclonal antibody, clone 123C3.D5, Bio-SB, USA) while the tumor cells were negative for pancytokeratin (1:200 dilution, mouse monoclonal antibody, clone A1A3, SycTek, USA) and LCA (1:500 dilution, mouse monoclonal antibody, clone 2B11 & PD7/26, Bio-SB, USA). A final pathological diagnosis of malignant melanoma with neuroendocrine differentiation was rendered. Subsequently, a biopsy from the liver lesion was also carried out which displayed tumor with similar morphological features. Electron microscopic examination was performed in our institute using paraffin-embedded tissue material. One to two millimeter of tissue was deparaffinized in xylene followed by graded alcohol series and was immersed in 2.5% glutaraldehyde overnight. After buffer wash, secondary fixation in 1% osmium tetroxide for 2 h at 4 °C was done, again followed by washing in buffer to remove excess fixative. Dehydration was performed by passing the tissue through increasing concentrations of alcohol. After dehydration, the tissue was infiltrated and embedded using epoxy resin. Polymerization was achieved by keeping the embedded blocks at 50-60 °C for 12-24 h. Ultrathin sections were then cut and stained by uranyl acetate and lead citrate. Ultrastructural examination revealed the presence of both melanosomes (Fig. 4a) and neurosecretory granules (Fig. 4b) in the tumor cells further confirming the pathological diagnosis. No skin lesions were found on re-examining the patient following this diagnosis, before labeling it as primary urethral MM with neuroendocrine differentiation. The patient was started on palliative (dacarbazine based) chemotherapy due to lack of feasibility of immunotherapy. Later, the patient defaulted treatment and was lost to follow-up.

## Discussion

MM is one of the rarest tumors of the female urethra accounting for 0.2% of all malignant melanomas and 4% of all urethral cancers. Most MMs of the female urethra are located at the meatus or in the distal urethra [5]. No clear evidence exists regarding the genetic predisposition or any specific agent as a risk factor for urethral melanoma. The most common presenting symptom is a urethral protruding mass followed by bleeding, hematuria, dysuria, or incontinence. However, obstruction is rare and acute retention as a presenting symptom has not been reported earlier. While most reported urethral melanomas are small, this case had a large 5 cm mass at presentation which is the second-largest among the reported cases so far [6].

MM is known to exhibit divergent differentiation towards different lineages which can be identified by immunohistochemistry. Neuroendocrine differentiation in MM involving various other sites has been reported in nine cases till present which comprises of two cases of primary cutaneous, one nasal, four primary choroidal, one anal canal, and one uveal melanoma [7–10]. However neuroendocrine differentiation in MM of the urethra represents a very rare phenomenon, and to the best of our knowledge, no such case has been documented in the literature.

The tumor cells show immunopositivity for both melanocytic markers (HMB-45 and Melan-A) and neuroendocrine markers (synaptophysin and chromogranin). Although “anomalous” or “aberrant” immunoreactivity of synaptophysin has been seen by RC Romano et al., but chromogranin is a specific marker for neuroendocrine differentiation and aberrant expression is exceptionally rare which would typically remains confined to a minority of cells only [11]. However, our case showed diffuse and strong chromogranin expression which pointed towards a neuroendocrine differentiation rather than an aberrant expression.

Differential diagnosis considered in the present case was pigmented neuroendocrine carcinoma/tumor which has been rarely reported at sites like the lung and pancreas. The tumor in such cases showed neuroendocrine morphology along with the presence of brown-black pigment in the cytoplasm of neoplastic cells. However, ultrastructural examination revealed the pigment to be lipofuscin or neuromelanin which were electron-dense granules deposited in an opaque matrix admixed with lipid vacuoles. Definite melanosomes or premelanosomes were not identified in any of them [12] as opposed to our case. In most of the reported cases of melanoma with neuroendocrine differentiation, small cell morphology or poorly differentiated tumor morphology prompted the usage of a panel of neuroendocrine markers. In our case, also, the small cell morphology was apparent leading to consideration of a differential of small cell carcinoma; however, subtle findings of conspicuous nucleoli at places and melanin pigment in few cells were also identified. Presence of overlapping morphological features compelled us to perform immunohistochemistry for both melanocytic and neuroendocrine markers. The unambiguous neuroendocrine features were confirmed at both immunohistochemical and ultrastructural levels and justified the diagnosis of MM with neuroendocrine differentiation in our case. The prognostic and therapeutic significance of such aberrations is uncertain.

Because of lack of visibility and non-specific symptoms during the early stage of the tumor, the diagnosis of urethral MM is often delayed and thus it carries a worse prognosis. Around 50% of the patients have inguinal lymph node metastasis at the time of diagnosis and distant metastasis occurs in one-third of them. Our case also had both lymph node and distant metastases at the time of presentation itself. Wide local excision with 2–2.5 cm margins and sentinel lymph node biopsy is an option for patients with localized disease. For metastatic and locally advanced disease, surgery is not recommended. Instead, chemotherapy and immunotherapy may be used in such cases, of which immunotherapy is preferred [1, 6]. Our patient had widespread metastasis, so chemotherapy was initiated.

## Conclusions

Neuroendocrine differentiation in MM is a rare phenomenon, which can be easily missed by unwary pathologists and can lead to diagnostic uncertainty. With the help of advertently chosen immunohistochemical panel and the input of electron microscopy, the nature of the cellular differentiation of these tumors can be picked up which leads to a correct diagnosis.

## Data Availability

Not applicable

## Abbreviations

MM: Malignant melanoma
CT: Computed tomography
H&E: Hematoxylin and eosin
LCA: Leucocyte common antigen
